# Extracts of pine bark (*Pinus sylvestris*) inhibit *Cryptosporidium parvum* growth in cell culture

**DOI:** 10.1007/s00436-021-07220-w

**Published:** 2021-07-16

**Authors:** Berit Marie Blomstrand, Heidi Larsen Enemark, Øivind Øines, Håvard Steinshamn, Inga Marie Aasen, Karl-Christian Mahnert, Kristin Marie Sørheim, Spiridoula Athanasiadou, Stig Milan Thamsborg, Ian David Woolsey

**Affiliations:** 1 Norwegian Centre for Organic Agriculture, Tingvoll, Norway; 2 Department of Animal Health and Food Safety, Norwegian Veterinary Institute, Oslo, Norway; 3 Division of Food Production and Society, Grassland and Livestock, Norwegian Institute of Bioeconomy Research, Tingvoll, Norway; 4 SINTEF Industry, Biotechnology and Nanomedicine, Trondheim, Norway; 5 The Norwegian Institute of Wood Technology, Oslo, Norway; 6 Animal and Veterinary Sciences, Scotland’s Rural College, Edinburgh, UK; 7 Department of Veterinary and Animal Sciences, University of Copenhagen, Frederiksberg, Denmark

**Keywords:** *Cryptosporidium parvum*, Pine bark, Condensed tannins

## Abstract

The widespread apicomplexan parasite *Cryptosporidium parvum* is responsible for severe gastrointestinal disease in humans and animals. The treatment options are limited, and the efficacy of available drugs is low. Bark contains condensed tannins (CT), which are bioactive compounds previously shown to inhibit parasite development. Here, we examined the anti-cryptosporidial properties of bark extract of Scots pine (*Pinus sylvestris*) against *C. parvum* by means of an in vitro growth inhibition test. We hypothesised that bark extracts would have dose-dependent inhibitory effects on the development of *C. parvum* in cell culture.

Bark extracts from Scots pine extracted with acetone, methanol, and water as solvents were investigated using human colorectal adenocarcinoma cells infected with *C. parvum*. Oocysts were inoculated onto the cell monolayer and bark extract was added at seven different concentrations. Parasite growth inhibition was quantified by qPCR.

The acetone and methanol extracts demonstrated a sigmoid dose-dependent inhibition of *C. parvum*. The IC_50_ values were 244.6 and 279.1 µg dry matter extract/mL, and 25.4 and 24.1 µg CT/mL, for acetone and methanol extracts, respectively. The IC_50_ for both extracts were similar, both with regard to the dry matter concentration of each extract and to CT concentrations.

Given the limited treatment options available for *Cryptosporidium* spp., the evidence generated in our study encourages further investigation into the in vitro and in vivo effects of pine bark extracts against *C. parvum.*

**Supplementary Information:**

The online version contains supplementary material available at 10.1007/s00436-021-07220-w.

## Introduction

*Cryptosporidium parvum* is an apicomplexan parasite with a worldwide distribution and a high zoonotic potential. Oocysts excreted in the faeces are immediately infective. Transmission is via the faecal-oral route, directly from host to host, or indirectly via the environment, by ingestion of contaminated food or water (Kosek et al. 2001) or possibly via insect vectors (Graczyk et al. 2003). Globally, cryptosporidiosis is a significant cause of diarrhoeal disease in humans and animals, and *C. parvum* is one of the most frequently diagnosed agents, with possible fatal consequences in children and immunocompromised individuals (Innes et al. 2020). *Cryptosporidium* spp. may lead to chronic joint pain, fatigue, and post-infectious irritable bowel syndrome in humans (Carter et al. 2019). Similarly, *C. parvum* causes diarrhoea, loss of condition, and reduced growth rate in new-born calves, lambs, and other vertebrates (Smith 2008). Cryptosporidiosis in Norway is generally believed to be underdiagnosed (Nygård et al. 2003). A survey between 2001 and 2003 demonstrated *C. parvum* infection in 53% of the dairy farms included in the study (*n* = 136) (Hamnes et al. 2006a), and a study on wild cervids completed in the same time period concluded that *C. parvum* is widespread among the cervid population in Norway (Hamnes et al. 2006b). An outbreak of human cryptosporidiosis in Norway was first reported in 2006 and was related to contamination from calves (Robertson et al. 2006). In 2011 and 2014, two additional animal associated human outbreaks were reported (Rimšelienė et al. 2011; Lange et al. 2014).

The options for treatment and prevention of cryptosporidiosis in both animals and humans are limited and, in many cases, suboptimal. Presently, there are to our knowledge only two moderately effective pharmaceuticals applied for metaphylactic treatment of calves: halofuginone lactate and paromomycin (The European Commission 2020; US food and drug administration 2020). Nitazoxanide is the only licenced pharmaceutical for use in humans, and the drug has limited effect in immunocompromised patients. Currently, there are no vaccines against *C. parvum*, and the robust oocysts are highly resistant to the most commonly used disinfectants (Innes et al. 2020).

There is evidence that dietary plant secondary metabolites (PSM) possess antiparasitic properties, both in vitro and in vivo (Anthony et al. 2005; Hoste et al. 2015). For instance, condensed tannins (CT) have proven antiparasitic effects (Dhakal et al. 2015; Desrues et al. 2016b; Spiegler et al. 2017). Few studies have tested the activity of different PSM against *C. parvum* (Sreter et al. 1999; Shahiduzzaman et al. 2009; Teichmann et al. 2012, 2016; Gaur et al. 2018; Woolsey et al. 2019b), and even fewer have tested CTs against *C. parvum* in animals (Kim and Healey 2001; Derbakova et al. 2016). To our knowledge, bark extracts from pine (*Pinus sylvestris*) have not previously been tested systematically against *C. parvum* in cell cultures in vitro*.*

The current insufficient treatment options against cryptosporidiosis and the possibility to exploit large amounts of excess bark from the Norwegian forest industry offer a strong incentive to further explore novel approaches to control cryptosporidiosis including bioactive compounds from bark extracts. In this study, we assessed the anti-cryptosporidial properties of bark extract of Scots pine against *C. parvum* by means of an in vitro growth inhibition test (Slifko et al. 1997; Woolsey et al. 2019a). We hypothesised that bark extracts with high CT concentration would have dose-dependent inhibitory effects on the intracellular development of *C. parvum* in cell culture.

## Materials and methods

### Parasite material

*Cryptosporidium parvum* oocysts (Iowa strain) purchased from Bunch Grass Farm (ID, USA) harvested from suckling calves and isolated by sucrose gradient centrifugation within 14 days prior to delivery to the institute were stored at 2 × 10^7^ oocysts/mL in 50 mL phosphate-buffered saline (PBS) with penicillin 1000 IU and streptomycin 1000 µg (2–7 °C). The viability of the oocysts was assessed within a week before the assays by staining oocysts in wet mounts with 4’,6-diamidino-2-phenylindole (DAPI) and propidium iodide (PI) (Campbell et al. 1992; Petersen and Enemark 2018). The oocysts (≥ 100) were counted using immunofluorescence microscopy, and the viability percentage was calculated (90%). This percentage was taken into account when calculating the concentration of viable oocyst in the stock solution.

The oocyst concentration was assessed within a week prior to the trial by diluting 10 µL stock solution with 990 µL PBS. Subsequently, 10 samples of 5 µL diluted stock solution were stained with Crypt-a-Glo (Waterborne Inc, LA, USA) according to the product instructions and counted using an immunofluorescence microscope fitted with a fluorescein isothiocyanate (FITC) filter.

### Cell culture

Human ileocecal colorectal adenocarcinoma (HCT-8) cells (ECACC, Salisbury, UK) (Upton et al. 1994; Joachim et al. 2018) were maintained as described by Woolsey et al. (2019a). A cell subcultivation was performed twice a week, when 80% confluent, using trypsin/EDTA (Sigma Aldrich, MO, USA). The cell cultures were incubated at 37 °C, 5% CO_2_ and 100% humidity. HCT-8 cells (2 × 10^5^ per well) were seeded onto Nunclon® 96-well plates (Sigma Aldrich, MO, USA) and incubated till 80% confluence (24 h).

### Bark extraction and determination of the concentration of condensed tannins (CT)

Bark from *P. sylvestris* was ring debarked and collected in a sawmill in eastern Norway (Bergene Holm AS, Kirkenær) in March 2017 and stored at − 20 °C until use. The bark was milled to chips of 0.5–2 cm in a hammer mill (Schutte Mini Mill, Buffalo, NY, USA), freeze-dried, and ground to particle sizes of approximately 2 mm in a coffee grinder. Aqueous acetone (70%), aqueous methanol (80%), and water were used as solvents producing three different extracts, PS-Ac, PS-Me, and PS-H2O, respectively. PS-Ac and PS-Me were prepared by adding 200 mL solvent to 10 g fine-ground bark, in an ultrasonic cleaning bath at full power (60 W) for 20 min (temperature increasing from 20 to 30 °C). For PS-H2O, 100 ml water was added to 10 g fine-ground bark, the extract was separated, and the procedure repeated. The temperature was 65 °C, and the time was 1 h for each of the two steps. The extracts were isolated by centrifugation and filtration (Whatman no 1) using vacuum suction. The organic solvents (acetone or methanol) were removed by evaporation (Rotavapor, 38 °C) before freezing and freeze-drying. For PS-H2O, the combined extracts were concentrated in a vacuum centrifuge at 65 °C (Thermo Scientific Savant SC250EXP SpeedVac Concentrator) to approximately 50% of the volumes, before freezing and freeze-drying. The dried extracts were stored at − 20 °C until further use.

Total CTs were quantified by the butanol-HCl assay. Freeze-dried extracts were dissolved in methanol (80% in water) and analysed with cyanidin-HCl as standard (Grabber et al. 2013), using the conventional reagent without acetone, 2.5 h, and absorbance reading at 545 nm.

To determine the relative monomer composition, the mean degree of polymerisation (mDP), and cis–trans-ratio, the methanol extracts were thiolysed with cysteamine hydrochloride and analysed by HPLC (Bianchi et al. 2015) using an Ascentis Express C18 column (15 cm × 2.1 mm, 2.7 µm, Supelco) and a flow rate of 0.3 mL/min.

Immediately prior to use, the dried extracts were dissolved in 100% dimethyl sulfoxide (DMSO), vortexed > 1 min, and diluted down to final extract concentrations of 300 µg/mL, 250 µg/mL, 200 µg/mL, 150 µg/mL, 100 µg/mL, and 50 µg/mL dry matter extract in 1% DMSO using maintenance medium (MM; RPMI-1640 (Biowest, France) supplemented with 5% v/v bovine foetal serum, 5% v/v horse serum, 1 mM sodium pyruvate, penicillin 100 U/mL, streptomycin 100 µg/mL, and amphotericin B 0.25 µg/mL (all from Sigma Aldrich, MO, USA)).

### Parasite inoculation onto cell monolayer and addition of extract

Before inoculation, the oocysts went through an excystation protocol as described earlier (Slifko et al. 1997; Woolsey et al. 2019a): 2 × 10^6^ viable oocysts (120 µL) and 2 × 10^6^ inactivated oocyst (IO) solution (120 µL stock solution previously incubated at 70 °C for 30 min) were suspended in bleach (120 µL 5.35% sodium hypochlorite and 960 µL MilliQ water) for 10 min on ice, and then centrifuged at 4000 × g for 4 min at 4 °C. The supernatant was aspirated, and 14 mL MilliQ water was added to each of the suspensions. The solutions were vortexed for 10 s and spun again (4000 × g, 4 min, 4 °C). The supernatant was aspirated down to 200 µL and the oocysts were re-suspended in 9.8 mL pre-warmed MM (37 °C), which gave a final oocyst concentration of 2 × 10^5^ oocysts per millilitre.

Oocyst solution (100 µL) was added to each monolayer well with a multichannel pipette with IO and MM as controls (*n* = 3). The plates were incubated for 4 h (37 °C, 5% CO_2_, 100% humidity), subsequently the wells were washed 5 min with PBS, and bark extracts were added to the live oocyst wells at the extract concentrations as mentioned above (biological repeat, *n* = 3 for each concentration). DMSO (1%) in MM was added to the negative MM controls and the IO wells, and paromomycin (500 µg/mL) in MM was added as positive control (*n* = 3) (see supplementary material for a plate overview). Each plate was duplicated.

### Cell viability

After 44-h incubation (37 °C, 5% CO_2_, 100% humidity) (Slifko et al. 1997), the cell viability was determined using an integrated non-destructive water-soluble tetrazolium salt assay (WST-1 cell proliferation assay kit; Roche, Basel, Switzerland): MM was aspirated from the wells and 10-µL WST-1 10% working solution was added to each well. The plates were incubated for > 30 min and the optical density was read at 450 nM (OD_450_). The plates were read every 15 min until all negative control wells had reached an OD_450_ value of > 1. Monolayers were considered viable if OD values in the extract wells were > 75% of the negative controls (Teichmann et al. 2012).

### DNA extraction

The wells were washed (3 × 5 min) with 100 µL PBS (37 °C), and 20 µL proteinase K and 180 µL ATL buffer (Qiagen, Hilden, Germany) were added to each well. Then, the plates were incubated (56 °C, 90 min) and the content of each well was aspirated into 1.5 mL Eppendorf tubes. DNA extraction was performed by means of QIAcube DNeasy® blood and tissue kit (Qiagen, Hilden, Germany) (tissues and rodent tails protocol, elution volume: 200 µL).

### qPCR assay

The growth of *C. parvum* in the cell cultures was assessed by measuring the amount of *C. parvum* specific DNA. Quantitative polymerase chain reaction (qPCR) was performed using 18S primers (Morgan et al. 1997) (Cryp18S_Frt 5’-AGTGACAAGAAATAACAATACAGG and Cryp18S_Rrt 5’-CCTGCTTTAAGCACTCTAATTTTC-3’) with hydrolysis probe (Amann et al. 1990; Keegan et al. 2003) (Integrated DNA Technologies, Coralville, IA, USA). Quantitative PCR reactions were conducted in a total volume of 25 µL containing 3 µL of each forward and reverse primers, 0.6 µL probe, 12.5 µL 2 × Brilliant III Fast Master Mix (Agilent Technologies, Santa Clara, CA, USA), 5.4 µL dH_2_O (nuclease-free water, Integrated DNA Technologies, Coralville, IA, USA), and 0.5 µL DNA template. Reactions were run on 96-well white-bottomed qPCR plates (Bio-Rad, Hercules, CA, USA).

From each biological replicate plate, extracted DNA from each biological repeat (*n* = 3) was run in duplicate (2 technical repeats) on the qPCR plate. DNA template from a separate oocyst titration study (cell monolayer infected with 5 × 10^4^–50 oocysts/well, 2 biological and 2 technical repeats per biological repeat per concentration) was added to each qPCR run to serve as DNA standards (Woolsey et al. 2019a).

The cycling conditions were initially 95 °C for 2 min followed by 44 cycles (95 °C for 10 s, 58 °C for 10 s, 72 °C for 20 s) on C1000 Touch (BioRad, Hercules, CA, USA). The definition of the cycle threshold was set at the beginning of the rise of the log-linear phase, and a quantification cycle (Cq) above 40 cycles was defined as negative based on the non-template controls included on each qPCR plate (Bustin et al. 2009). To analyse the amplification curves, BioRad CFX manager v3.1 software was used and the Cq values from the standard templates with known oocyst number were used to calculate the DNA level of the test templates. The data used in the subsequent analyses were defined as *C. parvum* DNA quantity relative to the DNA standard templates (DNA_rel_).

### Statistics

qPCR standard curves were produced in BIORAD CFX Manager v3.1. For each extract (PS-Ac, PS-Me, and PS-H2O), data used for statistical analyses was collected from two independent parasite growth inhibition assays with three biological repeats per extract concentration and two technical repeats per well per assay. By plotting the seven extract concentrations as independent variables and DNA_rel_ as dependent variable, it appeared that the relationship was inverse sigmoid for PS-Ac and PS-Me. We therefore analysed the data using a non-linear regression procedure (NLIN) in SAS (SAS release 9.4, SAS Institute, Cary, NC), and applied this to calculate the half maximal inhibitory concentration (IC_50_) for both PS-Ac and PS-Me. Two-sided *p* values of ≤ 0.05 were considered significant. The results from the PS-H2O assay were plotted into a simple (x,y)-plot and fitted a linear regression model.

## Results

### Characterisation of bark extracts

The extract DM yield per gram bark was 55, 47, and 54 mg/g for PS-Ac, PS-Me, and PS-H2O, respectively. The mean CT concentrations of PS-Ac, PS-Me, and PS-H2O were 95.3 ± 3.0, 90.9 ± 2.2, and 33.3 ± 2.5 mg/g DM extract (± s.d.) (9.5, 9.1, and 3.3%), respectively. The final CT concentrations in the dilutions thus ranged from 4.8 to 28.6, 4.5 to 27.3, and 1.7 to 10.0 µg/mL in PS-Ac, PS-Me, and PS-H2O, respectively. For the methanol extract, the PC:PD ratio was > 99:1, cis:trans ratio 79:21, and mDP 5.8.

### Cell viability

HCT-8 monolayer viability was within threshold limits of 75% of the negative controls in all wells on all plates except for two wells (yielding 70.0 and 73.8% of the negative controls), which were excluded from the later calculations.

### C. parvum growth inhibition assay

Based on model property and the result of the statistical analysis (model mean square error and the value of the parameter estimates relative to their respective standard error), the following model (Model 1) was chosen for the extracts PS-Ac and PS-Me when testing the extract dry matter (DM) concentrations for anti-cryptosporidial effect (*p* < 0.01):

$${\mu }_{y}\left(x\right)=\frac{\gamma }{1+{\left(\frac{x}{\delta }\right)}^{\theta }}$$

**Fig. 1:**
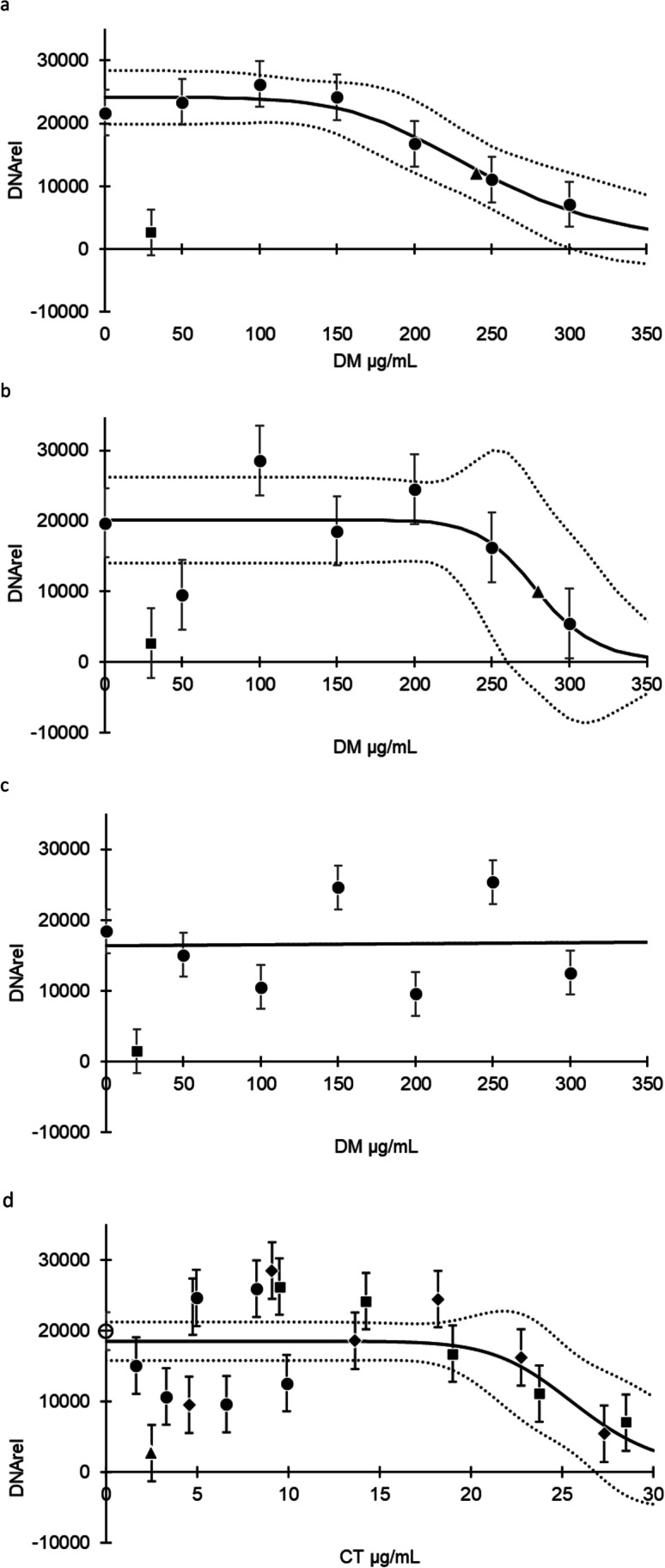
Inhibition of *Cryptosporidium parvum* development (black line) with increasing dry matter (DM) (**a**–**c**) or condensed tannins (CT) (**d**) concentrations from acetone (**a**), methanol (**b**), and water (**c**) based bark extracts from Scots pine (*Pinus sylvestris*). Black circles, mean DNA content relative to the standards (DNA_rel_) of observed values for each extract dose. Square, positive control (paromomycin). Triangle, half maximal inhibitory concentration (IC_50_). Error bars on observed values represent standard error of the mean (SEM). Area between dashed lines: 95% confidence interval. (**d**) DM concentrations was converted to CT concentrations, and all extracts were merged (PS-CT_m_). PS-CT_m_ exhibited a negative correlation with DNA_rel_. The symbols are mean of observed values of PS-Ac (squares), PS-Me (routes), and PS-H2O (black circles). The open circle displays the mean of the negative controls and the triangle the positive control

${\mu }_{y}(x)$ is the DNA_rel_ of a given value $x$, $x$ is the extract DM concentration, $\gamma$ is the value of ${\mu }_{y}(x)$ when no extract is applied ($x$ = 0), $\delta$ is the $x$ value at the inflection point (concentration of the strongest decline in DNA_rel_), which is equivalent to IC_50_, and $\theta$ describes the slope of the curve, i.e., the reduction in DNA_rel_ per unit increase in extract (Table 1). Based on the calculation, we found a dose-dependent relationship between the extract DM concentration (PS-Ac and PS-Me) and DNA_rel_ (Fig. 1a and 1b).

**Table 1:** Parameter estimates describing inhibition related to dry matter (DM) and CT concentrations

Parameter	Extract (DM)		Extract (CT)	Extract (CT)
	PS-Ac	PS-Me	PS-Me	PS-CT_m_
Model *p* value	< 0.0001	< 0.0001	< 0.0001	< 0.0001
$\gamma$	24,125.4 (1926.5)	20,103.8 (2755.8)	20,103.8 (2755.8)	21,632.5 (1448.6)
$\delta$ (IC_50_) (µg/mL)	244.6	279.1	25.4	26.2
$\theta$	5.2594 (2.3817)	14.6109 (15.1513)	14.611 (15.1514)	11.2822 (8.015)

Data converged when using the function ${\mu }_{y}\left(x\right)=\frac{\gamma }{1+{\left(\frac{x}{\delta }\right)}^{\theta }}$, where $x$ is the concentration, $\gamma$ is the value of ${\mu }_{y}(x)$ when $x$ = 0, $\delta$ is the $x$ value giving ${\mu }_{y}\left(x\right)=\frac{1}{2}\cdot {\mu }_{y}(0)$ (IC_50_), and $\theta$ gives the slope of the curve

We used the same principles to investigate the relationship between CT concentration and DNA_rel_. Data from PS-Me (CT) converged using Model 1, and its estimated parameters are presented in Table 1. The data from PS-Ac (CT) converged when using the following Model 2 (*p* < 0.0001):

#### $E(y)=\alpha \cdot {e}^{-\beta \cdot {e}^{k\cdot x}}$.

$E(y)$ is the oocyst concentration of a given value $x$, $x$ is the extract CT concentration, α and *β* describe the DNA_rel_ when no extract was applied ($x$ = 0), and *β* and $k$ form the slope of the curve. Table 2 lists the estimated parameters for the PS-Ac CT curve.

**Table 2:** Parameter estimates describing inhibition related to CT concentration for PS-Ac

Parameter	Extract (CT)
	PS-Ac
Model *p* value	< 0.0001
$\alpha$	24,674.7 (2828.3)
*β*	0.0114 (0.026)
$k$	0.1713 (0.0865)
IC_50_ (µg/mL)	24.1

Data converged when using the function ${\mu }_{y}(x)=\alpha \cdot {e}^{-\beta \cdot {e}^{k\cdot x}}$, where $x$ is the CT concentration; $\alpha$ and *β* give the oocyst level when $x$=0; and *β* and $k$ describe the slope of the curve. IC_50_ is the half maximal inhibitory concentration for each extract

The IC_50_ for PS-Ac and PS-Me extracts were estimated to 244.6 and 279.1 µg DM/mL (24.1 and 25.4 µg CT/mL), respectively.

For PS-Ac, DNA_rel_ values were not significantly different from the positive control for concentrations higher than 200 µg/mL (*P* > 0.05). For PS-Me, this was observed in concentrations above 250 µg/mL (*P* > 0.05).

The results from the PS-H2O assay could not be fitted in any reasonable way by any of the sigmoid models we tried. A simple (x,y) plot of the observed data together with a fitted linear model shows no statistically significant relationship between PS-H2O extract concentration and DNA_rel_ (Fig. 1c).

The CT data from all three extracts were merged (PS-CT_m_) and the relationship between PS-CT_m_ and DNA_rel_ was estimated with extract as random variable. The data converged using Model 1, and estimated parameters can be seen in Table 1. We found a dose-dependent negative correlation between the PS-CT_m_ concentration and DNA_rel_ (Fig. 1d). The IC_50_ for PS-CT_m_ was estimated to 26.2 µg CT/mL.

## Discussion

This study revealed a dose-dependent anti-cryptosporidial effect of acetone and methanol bark extracts (PS-Ac and PS-Me) from *P. sylvestris*. Our findings support the results of other studies addressing the possible effects of pine extracts against protozoa. Kim and Healey (2001) demonstrated in vivo that ethanol extracted bark extracts from *Pinus pinaster* reduced the oocyst shedding and improved the overall health of mice infected with *C. parvum*. Similarly, a study of goats infected with *Eimeria* spp. demonstrated that feeding pine (*Pinus densiflora*) needles significantly reduced the oocyst excretion compared to untreated controls. In the treated goats, oocyst excretion 10 days post-treatment was reduced by 93%, relative to the pre-treatment oocyst excretion (Hur et al. 2005). In vitro antiprotozoal effect of bark extract has also been shown against *Eimeria* spp. of poultry, where water extracted pine bark reduced the oocyst sporulation by 77.2–86.4% relative to untreated controls (Molan et al. 2009).

Importantly, we showed that both PS-Ac and PS-Me were, in the highest concentrations, no different from their respective positive controls. This shows that the extracts have an in vitro anti-cryptosporidial effect close to that of paromomycin, one of the few pharmaceuticals registered for use in animals.

Furthermore, merging results of PS-Ac and PS-Me, we described a dose-dependent relationship between CT concentration and DNA_rel_. Previous evidence is not conclusive on whether the CTs in the plant extracts are the compounds responsible for their antiparasitic activity. In some cases, the observed activity is closely linked to CT concentration (Novobilsky et al. 2011, 2013; Desrues et al. 2016a), while others have been unable to demonstrate such a link (Castañeda-Ramírez et al. 2017; Hernández-Bolio et al. 2018; Esteban-Ballesteros et al. 2019). Possible explanations to this divergence include the degree of purity of the extracts, and it is evident that bioactivity of CTs is strongly related to chemical structure, e.g., type of monomers, mDP and degree of galloylation (Mueller-Harvey et al. 2019).

In this trial, the extracts with the highest CT concentration also demonstrated the highest antiprotozoal effect; thus, it is likely that CTs play an important role in the interaction between parasites and extracts. Nevertheless, the possibility that other PSM, such as other polyphenols or non-phenolic compounds like sesquiterpene lactones, may also have a role in the activity observed in this study cannot be excluded as previously suggested (Barone et al. 2019).

In our study, PS-Ac exhibited anti-cryptosporidial effect at a lower dose but had a lower rate of reduction compared to PS-Me (cf.$\theta$, Table 1). Additionally, the IC_50_ was lower for PS-Ac than for PS-Me. The CT concentration of both extracts was similar and showed approximately the same inhibition of the parasitic development. Yet, it was not possible to fit the data generated from both extracts to the same model. This may support the theory that unknown, non-CT components present had a clear influence on the anti-cryptosporidial effect and/or that differences in chemical composition were related to the extraction method.

To our knowledge, IC_50_ values for pine bark extracts tested against *C. parvum* have not been calculated before. Our findings were in accordance with Williams et al. (2014) who tested purified and fractionated acetone pine bark extracts against *Haemonchus contortus* in a larval migration inhibition test and estimated IC_50_ to be 40.4 µg CT/mL. Molan (2014) found IC_50_ values of 69 µg CT/mL in a *Trichostrongylus colubriformis* larval development inhibition assay with purified acetone pine bark extracts containing 35% CT. It has previously been shown that a high mDP and low PC:PD ratio are of importance when it comes to the antiparasitic activity (Mueller-Harvey et al. 2019). PS-Ac and PS-Me had a low CT percentage, low mDP, and negligible amount of PDs, which was generally in accordance with previous studies (Matthews et al. 1997; Bianchi et al. 2015, 2019; Desrues et al. 2016a), yet the anti-cryptosporidial effect was significant with a low IC_50_. This supports the assumption that other components may have contributed to the inhibition of the development of *C. parvum*.

For PS-H2O, there was no statistically significant relationship between the extract DM concentration and DNA_rel_ (Fig. 1c). Barone et al. (2019), on the other hand, tested 51 *Lotus corniculatus* water extracts in vitro and found dose-dependent anthelmintic activity in several extracts, demonstrating that the use of water as a solvent does not preclude the extraction of active antiparasitic compounds. The lack of biological activity can be explained by the fact that we were not able to achieve a high enough concentration (DM or CT) or that the active components cannot be extracted using water as solvent.

Our results confirmed that acetone and methanol solvents have a higher ability to extract proanthocyanidins (CTs) from pine bark compared to water. This solvent-dependent difference in CT extractability is in accordance with Ramos et al. (2013), who found that water extracted bark extract from *P. sylvestris* had a high extract yield but a low concentration of total phenol and CT compared to extracts produced with methanol and acetone as solvents (Ramos et al. 2013). A possible reason for the low CT yield when using water as solvent is explained by Matthews et al. (1997) who describe direct linkages between non-extractable CTs and the cell wall matrix and weaker CT-CT linkages extractable with aqueous methanol but not with water. Despite the lack of anti-cryptosporidial effect of PS-H2O at the concentrations tested, it is important not to dismiss water extracts as inactive. Acetone and methanol are strictly regulated (Klima- og miljødepartementet 2004), highly flammable, toxic organic solvents potentially harmful to humans. Large scale production of bark extracts with the use of organic solvents is costly, and potential production sites are hard to come by. Water, on the other hand, is non-toxic to living organisms, leaves a lower environmental footprint and is less costly and easier to use for large scale production of extracts. We recommend further in vitro studies to test pine water extracts against *C. parvum* in higher concentrations.

Cryptosporidiosis is considered a neglected disease and reducing oocyst shedding in animals is an important One Health goal as it reduces the risk of disease in humans (Innes et al. 2020). If pine bark extracts can be used to combat livestock cryptosporidiosis, they may be an environmentally friendly alternative to the current pharmaceuticals and constitute a step towards minimising contamination of drinking water and subsequent human cryptosporidiosis.

## Conclusion

Both acetone and methanol extracts of *P. sylvestris* showed marked anti-cryptosporidial properties by inhibiting the development of *C. parvum* in HCT-8 cell cultures. The effect of the bark extracts was dose-dependent with IC_50_ values for CT almost similar between extracts (approximately 25 µg CT/mL). At the highest concentrations, the inhibitory activities were similar to that of existing drugs. A dose-dependent effect could not be confirmed for the water extract. Ultimately, in vitro testing should be followed by in vivo experiments with pine bark extracts to assess their applicability and relevance for disease control in animals. We also suggest further exploration of the anti-cryptosporidial effect of water extracts.

## Data and materials availability

The datasets generated during the current study are available from the corresponding author on reasonable request.

## Supplementary Information

Below is the link to the electronic supplementary material.

Supplementary file1 (DOCX 18 KB)
