# Supplementary material for: Extracts of pine bark (Pinus sylvestris) inhibit Cryptosporidium parvum growth in cell culture
Source: Parasitol Res. 2021 Jul 16;120(8):2919–27. doi: 10.1007/s00436-021-07220-w (PMC8370916; doi:10.1007/s00436-021-07220-w)
Supplement: Supplementary file 1 — Supplementary file1 (DOCX 18 KB) [file 436_2021_7220_MOESM1_ESM.docx]

Plate layout of the cell culture assays testing *Pinus sylvestris* (PS) bark extracts against *C. parvum*. PS-extract: bark extracts in six different concentrations, each extract on one plate and each plate run in duplicates. Each extract concentration was run in three biological repeats (B1-3), with two technical qPCR repeats on each biological repeat. Controls: IO (inactivated oocysts), blank (non-infected cell culture), negative control (*C. parvum* infected cell culture), positive control (*C. parvum* infected cell culture treated with paromomycin). MM: Maintenance medium only.

Font graphic: Arial 10

Software: Microsoft Excel for Microsoft 365 MSO (16.0.13801.20288)
